# Prevalence and reasons for electronic nicotine delivery systems modifications among U.S. youth, young adult, and adult users

**DOI:** 10.1038/s41598-025-08722-8

**Published:** 2025-07-09

**Authors:** Lucy Popova, Bai Cham, Zachary B. Massey, Thi Phuong Thao Tran, Mohammed M. Alqahtani, Robert T. Fairman, Ruiyan Luo, Scott R. Weaver, David L. Ashley

**Affiliations:** 1 School of Public Health, Georgia State University, P.O. Box 3995, Atlanta, GA 30302 USA; 2 Disease Control and Elimination Theme, Medical Research Council Unit The Gambia at the London School of Hygiene and Tropical Medicine, Atlantic Road, Fajara, The Gambia; 3 TSET Health Promotion Research Center, Stephenson Cancer Center, University of Oklahoma Health Sciences, Oklahoma City, OK USA; 4 Department of Health Promotion Sciences, Hudson College of Public Health, University of Oklahoma Health Sciences, Oklahoma City, OK USA; 5 Department of Respiratory Therapy, College of Applied Medical Sciences, King Saud bin Abdulaziz University for Health Sciences, Riyadh, Saudi Arabia; 6 King Abdullah International Medical Research Center, Riyadh, Saudi Arabia; 7 Department of Health Promotion and Physical Education, Wellstar College of Health and Human Services, Kennesaw State University, Kennesaw, GA USA; 8 Department of Respiratory Services, King Abdulaziz Medical City, Ministry of National Guard Health Affairs, Riyadh, Saudi Arabia

**Keywords:** Electronic nicotine delivery systems, E-cigarettes, Youth and young adults, Prevalence, Flavors, Risk factors, Epidemiology

## Abstract

This study evaluated the prevalence of various electronic nicotine delivery systems (ENDS) modifications among U.S. ENDS users and reasons for these modifications. We conducted a national survey of U.S. youth (13–17 years old, n = 553), young adults (18–29 years old, n = 634), and older adults (30 + , n = 760) who reported past 30-day ENDS use in 2021. The result showed that, in each age group, a large majority had engaged in at least one ENDS modification (youth, young adults, and older adults respectively: 84.3%, 84.1%, 76.9%), with modifications to e-liquid (68.1%, 61.2%, 48.7%) and coils (63.7%, 61.4%, 57.2%) being the most common. For some modifications, prevalence varied by age group and race/ethnicity. Participants endorsed various reasons for modifications, many related to accessing or enhancing the flavor of the aerosol. For youth, having heard about the modification was an important reason for trying a modification. Conversely, young adults and older adults frequently mentioned making the device last longer and saving money. ENDS modifications are widespread, but the reasons for modification differ between age groups. Some of these common modifications can lead to potential harm; they need to be regulated through product standards and marketing decisions, and ENDS users should be educated about potentially dangerous modifications.

**Supplementary Information:**

The online version contains supplementary material available at 10.1038/s41598-025-08722-8.

## Introduction

Electronic nicotine delivery systems (ENDS) or e-cigarettes have grown in popularity in the U.S.; in 2023, 10.0% of middle and high school students (approximately 2.80 million)^1^, and in 2021 (the latest national data available), 11.0% of adults 18–24 years old and 6.5% of adults 25–44 years old were current (past 30 days) ENDS users^2^. ENDS comprise various devices, from those closely resembling cigarettes to tanks and mods. While some types of ENDS, particularly tanks and mods, are designed to be modified, all ENDS can be modified by users. For this paper, we refer to modifications as those manipulations and changes to the product that are both intended and unintended by the manufacturer.

Like ENDS themselves, modifications are diverse, from e-liquid to hardware (e.g., replacing batteries); in essence, every part of the device can be modified, as we documented in our qualitative^3,4^ and content analysis^5,6^ studies. Some modifications might be relatively benign (e.g., replacing part of the device with the same authorized part), whereas other modifications might be harmful (e.g., rewiring a battery or adding different substances to e-liquid). Many modifications to ENDS or e-liquid change the delivery of chemicals to the user, which may alter their harm. For example, operating coils at higher voltage can result in combustion and exposure to higher levels of harmful and potentially harmful chemicals^7,8^. Thus, documenting modification practices is important, especially harmful practices that may injure users. Moreover, the FDA considers how users may modify their devices when deciding whether to allow products on the market (e.g., closed versus open systems)^9^, making data about modifications important for regulatory decision-making.

Research on the prevalence and reasons for ENDS modifications has been growing. Qualitative studies have examined ENDS users’ behavior and perceptions of ENDS modifications^3,4,10^. Content analysis of ENDS modification videos on YouTube found that most focused on providing how-to instructions^5^. Specific ENDS modifications have been explored, with several studies examining the use of other substances (predominantly cannabis) in ENDS through surveys^11–13^ and content analyses^14^. For instance, an online national survey of youth and young adults who used ENDS in the U.S. found that modifications were common, including modifications not intended by manufacturers (e.g., refilling liquids in devices not designed for refilling)^15^. However, there is currently no clear estimate of how common these behaviors are at the national level, or what proportion of ENDS users engage in various types of modifications. This study aims to begin filling this gap by surveying youth, young adult, and adult ENDS users on various modifications to ENDS, including reasons for the modifications.

## Methods

### Study sample and procedures

In 2021, we conducted a national survey of 2369 U.S. youth and adults (13 years or older) who reported past 30-day ENDS use among Ipsos’ KnowledgePanel, a probability-based web panel representative of the U.S., and Ipsos opt-in panels. KnowledgePanel members are recruited through address-based sampling, and households are provided access to the internet and computers when needed. Sampling of KnowledgePanel members was stratified by age group: youth (13–17 years), young adults (18–29 years), and older adults (30+ years). To recruit the youth, Ipsos contacted pre-identified KnowledgePanel households with children in the target age range and obtained parental consent. Study-specific post-stratification weights were computed using an iterative proportional fitting (raking) procedure to adjust for differential non-response. Age-stratified demographic distributions from the 2018–2019 Tobacco Use Supplement of the Current Population Survey (TUS-CPS) for adult participants (gender by age, race/ethnicity, region by metropolitan area status, education, household income) and from the 2020 National Youth Tobacco Survey (NYTS) for the youth sample (viz., gender by age, race/ethnicity) were used. Participants from the opt-in panel were aligned with the KnowledgePanel sample using a calibration weighting process based on the aforementioned geodemographic distributions and a common set of attitudinal/behavioral measures asked of both samples that have been found to differentiate nonprobability and probability samples.

Of 2369 participants completing the survey, 422 were excluded due to logical inconsistencies, data quality issues, or excessive speed (≤ 4 min), resulting in the final analytic sample of 553 youth, 634 young adults, and 760 older adults. The median survey length was 21 min; participants received a cash equivalent of $5. Georgia State University’s Institutional Review Board approved the protocol (H21422).

### Measures

These data are part of a larger project about ENDS modifications. Earlier qualitative interviews with ENDS users^3,4,16^ and analysis of ENDS modification videos on YouTube^5^ guided the survey development. We conducted cognitive testing with adult and youth ENDS users prior to administration.

#### ENDS modifications

Participants were asked whether they themselves had ever made modifications to e-liquid, coil, and battery, with specific modifications listed in Table 2 and answers of yes or no. Participants could endorse multiple modifications.

#### Reasons for ENDS modifications

For each specific modification a participant reported making, they were asked the reasons for making the modifications. The list of reasons is provided in Supplementary Tables 7–12, and responses were yes or no. Participants could select multiple reasons; they were not mutually exclusive.

#### Demographic characteristics

Participants reported age, sex, racial identification, ethnicity, and education level.

### Data analysis

All analyses were adjusted for the complex survey design and non-response. Different weights were applied for each age cohort, and hence, we conducted subpopulation analysis for each age cohort using their respective weighting. Weighted prevalence (with corresponding confidence intervals) of the different forms of modification, including e-liquid, coil, battery, pod, voltage, and building from scratch, were reported. We stacked weights of different age groups to assess whether the prevalences were significantly different across these age groups using Chi-square tests. Among individuals who reported at least one ENDS modification, we calculated the weighted mean and standard error for the number of modifications across six types of modifications. Significant differences in the average number of modifications across age groups were assessed using ANOVA test. Post hoc tests were also conducted with Bonferroni correction to perform pairwise comparisons between age groups.

## Results

### Participant characteristics

The mean ages were 15.4 years for youth, 23.5 years for young adults, and 47.4 years for older adults (Table 1). Males comprised 47% of youth, 62.3% of young adults, and 55.3% of older adults. Over 70% of adults identified as White and non-Hispanic, while nearly half of youth identified as Hispanic or non-White. Older adults reported an average of 22.5 days of ENDS use per month; youth and young adults reported lower averages of 10.2 and 15.7 days per month, respectively.

**Table 1:** Sample characteristics by age group.

Characteristics	Youth 13–17 years old *n* = 553	Young adults 18–29 years old *n* = 634	Older adults 30 + years old *n* = 760
	Weighted % (95% CI)	Weighted % (95% CI)	Weighted % (95% CI)
Age (years), Mean	15.4 (15.3–15.6)	23.5 (23.2–23.9)	47.4 (46.4–48.4)
Gender			
Male	47.0 (42.5–51.5)	62.3 (57.2–67.1)	55.3 (50.9–59.6)
Female	52.2 (47.6–56.7)	37.7 (32.9–42.8)	44.7 (40.5–49.1)
A different identity	0.8 (0.2–3.3)	0.0	0.0
Race/ethnicity			
White, non-Hispanic	51.5 (46.9–56.1)	72.8 (68.0–77.2)	80.4 (76.6–83.7)
Black, non-Hispanic	6.0 (4.3–8.3)	5.3 (3.5–7.8)	5.8 (4.0–8.4)
Other, non-Hispanic	4.8 (3.1–7.5)	3.9 (2.4–6.3)	3.4 (2.0–5.5)
2+ races, non-Hispanic	9.0 (6.4–12.5)	3.9 (2.3–6.5)	3.0 (1.7–5.1)
Hispanic, Latina or Latino, or Spanish origin	28.6 (24.2–33.5)	14.1 (10.9–18.1)	7.5 (5.6–9.9)
Education			
Less than high school	95.0 (92.5–96.8)	8.2 (5.6–11.8)	7.5 (5.1–11.0)
High school	–	39.8 (34.7–45.2)	31.0 (26.6–35.8)
Some college	5.0 (3.2–7.5)	37.1 (32.0–42.5)	40.1 (35.8–44.4)
Bachelor’s degree or higher	–	10.4 (7.9–13.4)	12.3 (10.2–14.7)
Master’s degree or higher	–	4.6 (3.2–6.4)	9.1 (7.2–11.4)
ENDS use			
Age of ENDS initiation (years), Mean	13.9 (13.7–14.1)	19.2 (18.8–19.6)	41.1 (39.9–42.3)
Number of days of ENDS use in the past 30 days, Mean	10.2 (9.3–11.1)	15.7 (14.4–17.0)	22.5 (21.6–23.4)
Smoking status^a^			
Never smoker	54.3 (48.6–59.9)	31.4 (26.1–37.4)	5.6 (3.8–8.1)
Former smoker	2.7 (1.3–5.4)	19.3 (14.8–24.8)	56.7 (52.2–61.2)
Current smoker	43.0 (37.5–48.8)	49.2 (43.3–55.2)	37.7 (33.4–42.2)

The n-size columns for each age group are unweighted. The estimate columns and confidence intervals for the estimate precents are weighted. 95% CI = 95% Confidence Interval.

^a^Current smokers are defined as individuals who currently smoke cigarettes every day or some days.

### Prevalence of ENDS modifications

The majority of ENDS users in all age groups have made some modifications, with 84.3% of youth, 84.1% of young adults, and 76.9% of older adults (*P* = 0.006) (Table 2). On average, youth reported making more modifications (M = 9.0) than young adults (M = 6.6) and adults (M = 5.0).

**Table 2:**
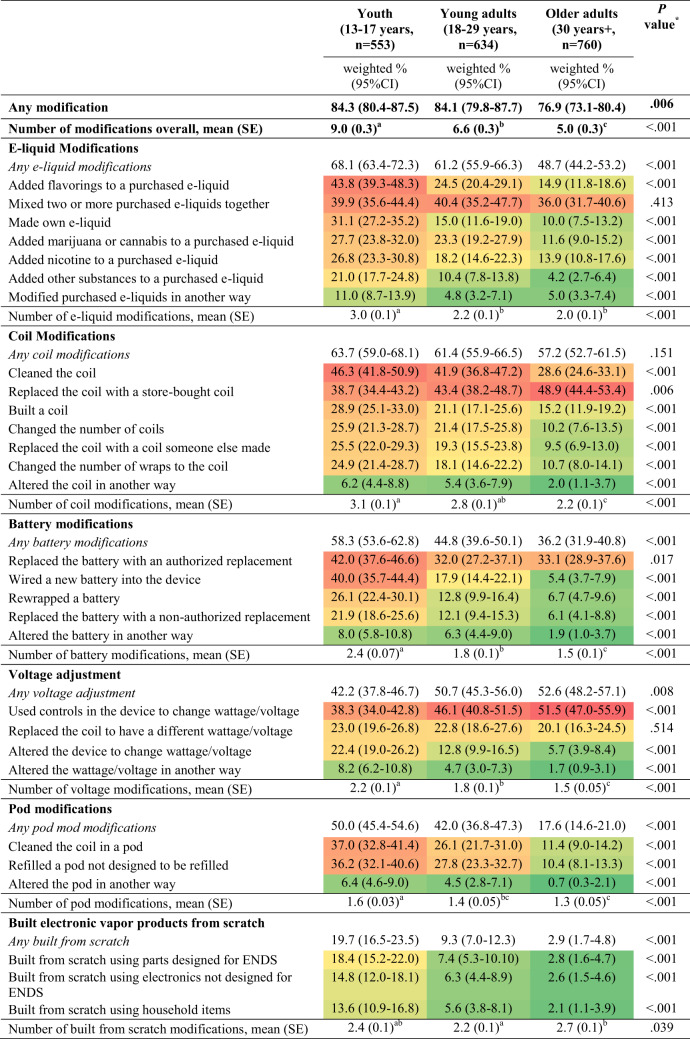
Prevalence of ENDS modifications by age group

Colors (heat map) indicate comparative prevalence of each modification across age groups, with darker reds indicating the highest prevalence and darker green the lowest. The heat map compares all individual modifications, but not “any modifications” (e.g., “any e-liquid modification”); those are left blank.

The estimates and confidence intervals for the estimated precent are weighted. 95% CI = 95% Confidence Interval.

Groups with non-overlapping confidence intervals are significantly different.

*Chi-square tests were used to test statistically significant differences in the prevalence across the three age groups using stacked weights. ANOVA was used to test significant differences in the average number of modifications across age groups.

^a,b,c^Groups with different superscripts are significantly different from each other, based on post hoc pairwise comparisons with Bonferroni correction.

The prevalence of any e-liquid modifications was highest among youth, lower among young adults, and lowest among older adults (*P* < 0.001). Among youth, 68.1% reported ever making any e-liquid modification, with the highest percent adding flavorings to a purchased e-liquid (43.8%), mixing two or more purchased e-liquids together (39.9%), and making their own e-liquid (31.1%). Similarly, among young adults, e-liquid modifications were reported by 61.2% of participants, with the most frequent ones being mixing two or more purchased e-liquids together (40.4%), adding flavorings (24.5%), and adding cannabis to a purchased e-liquid (23.3%). Among older adults, less than half (48.7%) have ever made e-liquid modifications, with the most frequent being mixing two or more purchased e-liquids together (36.0%).

There was no significant difference in the prevalence of any coil modifications among age groups (*p* = 0.151). Among youth, 63.7% said they ever modified coils, such as cleaning the coil (46.3%), replacing the coil with a store-bought coil (38.7%), building a coil (28.9%), and changing the number of coils (25.9%). Among young adults, 61.4% reported making coil modifications, with the highest percent reporting replacing the coil with a store-bought coil (43.4%), cleaning the coil (41.9%), and changing the number of coils (21.4%). Among older adults, 57.2% have modified coils, with the most popular modifications being replacing the coil with a store-bought coil (48.9%), cleaning the coil (28.6%), and building a coil (15.2%).

The prevalence of any battery modifications was highest among youth, lower among young adults, and lowest among older adults (*P* < 0.001). Among youth, 58.3% reported ever modifying ENDS batteries, mostly replacing the battery with an authorized replacement (42.0%) and wiring a new battery (40.0%). Among young adults, less than half (44.8%) reported making battery modifications, primarily replacing the battery with an authorized replacement (32.0%) and wiring a new battery (17.9%). Only 36.2% of older adults have ever modified ENDS batteries, and most were replacing the battery with an authorized replacement (33.1%).

In terms of wattage/voltage modification, 42.2% of youth, 50.7% of young adults, and 52.6% of older adults reported ever having modified the wattage/voltage (*P* = 0.008), mainly using controls in the device to change wattage/voltage (youth: 38.3%, young adult: 46.1%, and older adult: 51.5%).

Pod modifications were more common among younger participants (50.0% youth, 42.0% young adults) than older adults (17.6%) (*P* < 0.001). Approximately 20% of youth have built ENDS from scratch vs. 9.3% of young adults and 2.9% of older adults (*P* < 0.001).

### Prevalence of ENDS modifications by race and ethnicity for each age group

We examined whether the prevalence of modifications differed by race/ethnicity for each age group (Supplementary Tables 1–6). For e-liquid modifications, among youth, differences were significant for any e-liquid modification (*P* = 0.001), making own e-liquid (*P* < 0.001), adding nicotine to purchased e-liquid (*P* = 0.001), adding other substances to e-liquid (*P* = 0.001), and modifying e-liquid in other ways (*P* = 0.016). For all these, the highest percentage was among White non-Hispanic youth participants. Among young adults, differences were significant for making own e-liquid (*P* = 0.037), with the highest proportion reported by Hispanic participants. For adding cannabis to purchased e-liquid (*P* = 0.041), Black non-Hispanic participants reported the highest percentage. Among older adults, differences were significant for any e-liquid modification (*P* = 0.009), adding flavorings to a purchased e-liquid (*P* = 0.044), adding cannabis (*P* = 0.006) or other substances (*P* = 0.014) to a purchased e-liquid, and modifying e-liquid in another way (*P* = 0.002). For all but one of these modifications, the highest percentage was reported by Black non-Hispanic participants; Hispanic participants reported the highest percentage for adding other substances.

For coil modifications, among youth, differences were significant for building a coil (*P* = 0.004), replacing the coil with a coil someone else made (*P* < 0.001), changing the number of coils (*P* = 0.004), and changing the number of wraps to the coil (*P* = 0.006). For all these modifications, the highest percentage was reported by White non-Hispanic participants. Among young adults, there were no significant differences for coil modifications. The only significant difference among older adults was altering the coil in another way (*P* = 0.002), with Hispanic participants reporting the highest percentage (8.4%).

For battery modifications, among youth, differences were significant for any battery modification (*P* = 0.001), wiring a new battery into the device (*P* < 0.001), replacing the battery with an authorized replacement (*P* = 0.041), and rewrapping a battery (*P* = 0.012). Again, White non-Hispanic participants reported the highest percentages for all these modifications. Among young adults, the only significant difference was replacing the battery with a non-authorized replacement (*P* = 0.003), with Black non-Hispanic participants reporting the highest percentage. Among older adults, differences were significant for any battery modification (*P* = 0.013) with the highest percent reported by Black non-Hispanic participants, wiring a new battery into the device (*P* < 0.001) and rewrapping a battery (*P* = 0.020) with the highest percent for both reported by other non-Hispanic participants and replacing the battery with a non-authorized replacement (*P* < 0.001) and altering the battery in another way (*P* < 0.001), with the highest percent reported by Hispanic participants.

For wattage/voltage modifications, differences were significant for any wattage/voltage modification and all specific wattage/voltage modifications (*P* values ≤ 0.01) among youths, with the highest percentage reported by White non-Hispanic participants. No significant differences were found among young adults. Among older adults, differences were significant for altering the device to change wattage/voltage, with the highest percentage reported by Other non-Hispanic participants.

For pod modification, among youth, differences were significant for any pod modification (*P* < 0.001), refilling a pod not designed to be refilled (*P* = 0.001), and cleaning the coil in a pod (*P* < 0.001), with the highest percentage reported by White non-Hispanic participants. Among older adults, differences were significant for any pod modification (*P* < 0.001) and cleaning the coil in a pod (*P* < 0.001), with the highest percentage reported by Other non-Hispanic participants.

For building ENDS from scratch, among youth, differences were significant for building from scratch using electronics or household items not designed for ENDS, with the highest percentage reported by White non-Hispanic participants. Among young adults and older adults, building from scratch using parts designed for ENDS or using electronics or household items not designed for ENDS were significantly different by race/ethnicity. The highest percentage was reported by Black non-Hispanic participants among young adults and by Other non-Hispanic and Hispanic participants among older adults.

### Reasons for ENDS modifications

Frequencies of endorsed reasons for ENDS modifications are presented in Supplementary Tables 7–12, using a heat map. Average number of reasons endorsed was calculated only among people who made the specific modification and endorsed at least one reason for it. ENDS users reported making ENDS modifications for a variety of reasons. Youth seemed to endorse multiple reasons for the same modification at greater rates, followed by young adults and older adults. For example, for making their own e-liquid, almost all reasons were endorsed by at least half of the youth who made that modification, with “enhancing flavor” being endorsed by 90%. Among young adults, only 8 out of 15 (not including “other”) reasons for modification were endorsed by at least half of the participants, with enhancing flavor still being most commonly endorsed at 72%. In contrast, among older adults, only about a third of the reasons for making own e-liquid were endorsed by half or more of the participants; saving money was the top listed reason (82%).

Overall, enhancing flavor and trying new things were frequently selected reasons across all age groups. Economic reasons were also frequently selected, such as saving money and making the device or its parts last longer. Social reasons (e.g., others do it, having heard/read about it) were commonly endorsed by youth. Other reasons, such as changing the cloud size and nicotine hit/level, were less frequently ascribed.

## Discussion

In the U.S., across all age groups, ENDS users are making modifications to their devices. Among youth, 84.3% reported making at least one of the modifications we examined; these numbers were similar among young adults and older adults. Many of the most reported modifications were those designed by the manufacturers, such as using controls in the device to change wattage/voltage or replacing the coil with a store-bought coil. Others were in a neutral zone and might have been within the device manufacturing parameters, such as cleaning the coil. However, many of the highest-endorsed modifications, particularly among youth, were not intended by the manufacturers to be performed by users, such as adding flavorings to purchased e-liquids, cleaning the coil in a closed pod, and refilling a pod not designed to be refilled. One particularly dangerous modification is wiring a new battery, and 40% of youth ENDS users reported doing that. Youth were also more likely to replace the battery with an unauthorized replacement and to rewrap a battery. These battery modifications could lead to overheating, fire, or explosions, resulting in severe burns and injuries^17,18^.

Results of this study suggested that Black/Hispanic adults were more likely to carry out more dangerous modifications (i.e., battery modifications) compared to White adult ENDS users. Previous studies have not explored differences in ENDS modifications by race/ethnicity. These findings suggest that modification behaviors—and the risks associated with them—may not be evenly distributed across racial and ethnic groups. The observed differences highlight the importance of tailoring future educational interventions to ensure that they address the specific modification practices and risk perceptions of different subgroups of ENDS users. Therefore, it is essential to implement appropriate practices for effectively communicating potential risks associated with ENDS batteries to consumers and the general public, such as product labelling, hazard warnings, and instructions for safe use, where consumers may see warnings on packaging at times of purchase and use. Warning messages can be adapted to health campaigns and disseminated through multiple channels, such as school-based programs, clinician offices, and social media, where youth who cannot purchase ENDS may be exposed to information about the risk of modifying devices.

Enhancing flavor was among the most frequently endorsed reasons for modifications, especially among youth. Importantly, data were collected in an environment where ENDS flavors were mostly unrestricted except in some jurisdictions. This raises the question of whether flavor bans (such as those implemented in some states like California and New York) would increase modifications aiming to enhance flavor. On the one hand, since ENDS users frequently mix e-liquids of different flavors, not having those e-liquids readily available for sale might reduce this modifying behavior. On the other hand, if flavors are unavailable, ENDS users may engage in other manipulations with e-liquids to obtain the flavors they desire or expand the practice of adding other substances to the liquid. Research in jurisdictions that ban flavors is needed to answer this question.

Potentially dangerous modifications to e-liquids include adding cannabis and other substances not intended for vaping to e-liquids^19^. As expected, “getting high” was frequently endorsed as a reason for this modification (61–78%). However, these e-liquid modifications also were made for other reasons, mainly to enhance flavor (60–88%) and to try new things (55–78%). Ironically, 68% of youth who added other substances to e-liquids reported doing it to make it safer to vape. As our previous qualitative study showed, youth reported adding many other substances to e-liquids (such as honey or fruit juice) and did so primarily for flavor^10^. It is possible that some people who use ENDS might see these “healthy” substances as making the e-liquid safer. However, the same qualitative interviews revealed that some youth added other harmful substances to e-liquids, such as alcohol, cannabis, and cocaine. Modifying e-liquids with any substance not intended for vaping has the potential to alter the intoxicating effects of the product and the toxicant levels in ENDS emissions. Mixing alcohol with e-liquids, for instance, was associated with severe illness and death^20^. Beyond age-related trends, differences by race and ethnicity suggest that cultural, social, and access factors may shape modification behaviors. Higher rates of cannabis-related modifications among Black and Hispanic adults highlight the need for careful monitoring of these modifications.

The overall patterns of modifications showed notable differences between age groups that highlight public health concerns. For example, the prevalence of any battery and e-liquid modifications was highest among youth, lower among young adults, and lowest among older adults, with youth reporting potentially dangerous modifications, such as wiring a new battery, which can lead to over-heated batteries, fires, explosions, and injury^21^. Similarly, e-liquid modifications carry the risk for adverse health outcomes, including intentional or unintentional poisonings, production of toxic aerosol emissions, and acute intoxication. These are all modifications that could increase the harm of using ENDS products by youth (and young adults in some cases), and these data inform decision-makers about modifiable product features (e.g., accessible batteries) presenting a risk to consumers. Importantly, these socio-demographic differences do not imply that any one group is more prone to risk-taking, but rather that different populations may have different motivations, knowledge gaps, or social contexts that shape modification behavior. As such, future behavioral interventions should consider stratifying content or delivery channels by both age and race/ethnicity to ensure relevance and impact.

In terms of reasons for modifications, social influences (“others I know do it” and “heard/read about it”) were endorsed frequently by youth. Older participants endorsed social influence reasons less frequently. This is consistent with previous evidence about the influence of peers on youth behavior, including ENDS use^22,23^ and ENDS modification suggested by our study. Social influences on modification were more pronounced in youth compared to adults, possibly due to the sharing of products between adolescents themselves^24^. This behavior may also be motivated by a desire to save money among youth, which could explain why the “save money” reason was frequently reported among youth.

This study has several limitations. First, the youth sample was partially recruited through a non-probability panel. Second, although modifications examined were based on our earlier qualitative studies^3,4,16^ and content analysis of ENDS modification videos^5^, additional and newer modifications might have been missed. Third, we only inquired about ever-modification and did not measure the frequency or duration of modifications. Finally, although device type is likely to influence modification behaviors, we did not analyze modification patterns by device type in this manuscript. However, we recognize the importance of this variable and are examining it in a separate manuscript.

Future research should examine how often and how consistently ENDS users engage in different types of modifications, including whether certain behaviors (e.g., battery rewiring, cannabis mixing) are one-time experiments or routine practices. Studies should examine how local and state flavor bans and other regulations might impact modifications. Finally, future work should assess user knowledge about device safety and test targeted risk communication strategies to discourage high-risk modifications.

## Conclusion

ENDS modifications are common and undertaken for various reasons. Due to the severe health risks associated with modifications, the likelihood of ENDS users altering their products should be considered when developing ENDS product standards and during new product reviews. Additionally, it is essential to implement warnings and instructions for use to effectively communicate potential risks associated with ENDS modifications to consumers and the general public.

## Electronic supplementary material

Below is the link to the electronic supplementary material.

Supplementary Material 1

## Data Availability

Data dictionary and data to reproduce study findings can be obtained by contacting the corresponding author.

## Abbreviation

ENDS: Electronic nicotine delivery systems
