# Supplementary material for: Prevalence and reasons for electronic nicotine delivery systems modifications among U.S. youth, young adult, and adult users
Source: Sci Rep. 2025 Jul 9;15:24592. doi: 10.1038/s41598-025-08722-8 (PMC12238517; doi:10.1038/s41598-025-08722-8)
Supplement: Supplementary file 1 — Supplementary Material 1 [file 41598_2025_8722_MOESM1_ESM.docx]

**Supplementary Tables**

**Prevalence and reasons for electronic nicotine delivery systems modifications among U.S. youth, young adult, and adult users**

Lucy Popova, PhD^1^*; Bai Cham, PhD^1,2^; Zachary B. Massey, PhD^3,4^; Thi Phuong Thao Tran, PhD^1^; Mohammed M. Alqahtani, PhD^5,6,7^; Robert T. Fairman, PhD^8^; Ruiyan Luo, PhD^1^; Scott R. Weaver, PhD^1^; and David L. Ashley, PhD^1^

^1^ School of Public Health, Georgia State University, Atlanta, GA, USA

^2^Disease Control and Elimination Theme, Medical Research Council Unit The Gambia at the London School of Hygiene and Tropical Medicine, Atlantic Road, Fajara, The Gambia

^3^ TSET Health Promotion Research Center, Stephenson Cancer Center, University of Oklahoma Health Sciences, Oklahoma City, OK, USA

^4^Department of Health Promotion Sciences, Hudson College of Public Health, University of Oklahoma Health Sciences, Oklahoma City, OK, USA

^5^Department of Respiratory Therapy, College of Applied Medical Sciences, King Saud bin Abdulaziz University for Health Sciences, Riyadh, Saudi Arabia

^6^King Abdullah International Medical Research Center, Riyadh, Saudi Arabia

^7^Department of Respiratory Services, King Abdulaziz Medical City, Ministry of National Guard Health Affairs, Riyadh, Saudi Arabia

^8^Department of Health Promotion and Physical Education, Wellstar College of Health and Human Services, Kennesaw State University, Kennesaw, GA, USA

***Corresponding author:** Lucy Popova, PhD, School of Public Health, Georgia State University, P.O. Box 3995, Atlanta, GA 30302 ([lpopova1@gsu.edu](mailto:lpopova1@gsu.edu))

**Supplementary Table 1. Weighted prevalence of e-liquid modifications by race/ethnicity**

| **E-liquid Modifications** | **Weighted % (95%CI)** | | | | | ***P* value** |
| --- | --- | --- | --- | --- | --- | --- |
|  | **White, Non-Hispanic** | **Black, Non-Hispanic** | **Other, Non-Hispanic** | **Hispanic** | **≥ 2 Races, Non-Hispanic** |  |
| **Youths** **(13-17 years), n=553** |  |  |  |  |  |  |
| Any e-liquid modifications | 75.4 (70.2-79.9) | 48.1 (32.2-64.3) | 46.3 (25.4-68.6) | 69.0 (58.6-77.8) | 48.3 (31.7-65.4) | .0005 |
| Made own e-liquid | 44.8 (39.6-50.2) | 17.9 (8.0-35.2) | 4.3 (0.6-25.3) | 21.8 (14.7-31.4) | 4.6 (1.1-17.1) | <.0001 |
| Mixed two or more purchased liquids together | 44.9 (39.6-50.3) | 26.6 (14.7-43.3) | 37.7 (18.7-61.4) | 37.7 (28.6-47.8) | 28.2 (15.4-45.9) | .4953 |
| Added nicotine to purchased e-liquid | 37.0 (32.0-42.3) | 12.5 (5.1-27.6) | 9.3 (2.3-31.0) | 19.3 (12.7-28.1) | 11.5 (4.7-25.5) | .0005 |
| Added flavorings to a purchased e-liquid | 48.4 (43.0-53.8) | 30.0 (17.2-46.8) | 29.6 (13.4-53.5) | 45.9 (36.1-56.1) | 27.5 (15.4-44.1) | .1514 |
| Added marijuana or cannabis to a purchased e-liquid | 31.1 (26.4-36.3) | 17.8 (8.3-34.1) | 12.9 (4.1-34.1) | 27.6 (19.3-37.9) | 22.4 (11.5-39.0) | .5645 |
| Added other substances to a purchased e-liquid | 27.4 (22.9-32.3) | 15.7 (6.9-32.0) | 4.3 (0.6-25.3) | 18.6 (11.9-27.9) | 4.6 (1.1-17.1) | .0006 |
| Modified purchased e-liquids in another way | 16.4 (12.9-20.7) | 9.8 (3.1-27.1) | 0 | 4.7 (1.9-10.8) | 7.3 (2.3-20.7) | .0160 |
| **Young adults (18-29 years)**, **n=634** |  |  |  |  |  |  |
| Any e-liquid modification | 61.5 (55.2-67.5) | 67.9 (47.6-83.2) | 57.3 (33.4-78.2) | 59.5 (45.4-72.1) | 57.0 (30.1-80.3) | .9534 |
| Made own e-liquid | 14.5 (10.6-19.6) | 20.9 (9.6-39.8) | 1.4 (0.2-9.5) | 21.7 (12.9-34.0) | 3.6 (1.0-12.1) | .0374 |
| Mixed two or more purchased liquids together | 43.2 (36.9-49.6) | 24.4 (13.1-40.7) | 31.8 (14.6-56.1) | 31.0 (20.5-43.8) | 52.9 (27.5-76.9) | .4788 |
| Added nicotine to purchased e-liquid | 17.9 (13.7-23.1) | 15.9 (7.7-30.0) | 21.4 (9.3-42.0) | 21.4 (13.1-33.0) | 10.7 (3.1-31.3) | .3915 |
| Added flavorings to a purchased e-liquid | 21.7 (17.1-27.1) | 22.7 (12.3-38.0) | 35.3 (15.1-62.6) | 35.2 (24.6-49.8) | 26.6 (11.3-50.8) | .3693 |
| Added marijuana or cannabis to a purchased e-liquid | 20.0 (15.5-25.4) | 39.1 (21.2-60.5) | 21.0 (9.7-39.6) | 37.7 (25.7-51.3) | 12.7 (3.7-35.6) | .0406 |
| Added other substances to a purchased e-liquid | 9.7 (6.7-13.8) | 10.7 (4.3-24.1) | 3.4 (0.8-14.1) | 15.8 (8.4-27.5) | 10.9 (2.6-35.5) | .0822 |
| Modified purchased e-liquids in another way | 4.9 (3.0-7.6) | 3.5 (1.1-11.0) | 1.1 (0.1-7.8) | 6.9 (2.5-17.4) | 1.1 (0.1-7.5) | .9057 |
| **Older adults (30+ years), n= 760** |  |  |  |  |  |  |
| Any e-liquid modifications | 47.1 (42.1-52.2) | 76.3 (60.5-87.1) | 23.4 (7.6-53.2) | 56.1 (41.2-70.1) | 46.2 (22.3-72.0) | .0086 |
| Made own e-liquid | 8.0 (5.6-11.4) | 17.2 (6.8-37.5) | 21.8 (6.5-52.6) | 20.1 (10.2-35.9) | 9.4 (1.3-44.7) | .0860 |
| Mixed two or more purchased liquids together | 35.8 (31.0-40.9) | 52.3 (34.0-70.1) | 16.8 (4.1-49.0) | 36.1 (23.6-50.8) | 31.6 (13.6-76.0) | .5331 |
| Added nicotine to purchased e-liquid | 12.8 (9.6-17.0) | 26.2 (12.1-47.7) | 4.9 (1.1-19.5) | 24.7 (13.6-40.6) | 0 | .0510 |
| Added flavorings to a purchased e-liquid | 12.3 (9.2-16.3) | 34.3 (18.3-54.9) | 16.8 (4.1-49.0) | 27.7 (15.9-43.7) | 11.6 (2.3-41.9) | .0439 |
| Added marijuana or cannabis to a purchased e-liquid | 8.9 (6.5-12.2) | 32.0 (15.9-53.8) | 16.8 (4.1-49.0) | 23.2 (13.2-37.4) | 7.7 (2.3-23.0) | .0056 |
| Added other substances to a purchased e-liquid | 2.9 (1.6-5.2) | 7.4 (2.6-19.2) | 16.8 (4.1-49.0) | 11.3 (4.3-26.5) | 0 | .0141 |
| Modified purchased e-liquids in another way | 3.6 (2.1-6.0) | 16.7 (5.9-38.9) | 0 | 14.8 (6.8-29.4) | 0 | .0020 |

**Supplementary Table 2. Weighted prevalence of coil modifications by race/ethnicity**

| **Coil Modifications** | **Weighted % (95%CI)** | | | | | | ***P* value** |
| --- | --- | --- | --- | --- | --- | --- | --- |
|  | **White, Non-Hispanic** | **Black, Non-Hispanic** | **Other, Non-Hispanic** | **Hispanic** | **≥2 Races, Non-Hispanic** |  | |
| **Youths (13-17 years), n=553** |  |  |  |  |  |  | |
| Any coil modifications | 69.7 (64.3-74.7) | 53.7 (37.1-69.5) | 58.8 (36.1-78.2) | 59.1 (48.8-68.7) | 52.8 (35.5-69.5) | .3794 | |
| Built a coil | 37.1 (32.1-42.4) | 21.4 (10.6-38.4) | 21.0 (7.7-45.8) | 24.0 (16.5-33.6) | 6.6 (2.1-19.0) | .0035 | |
| Replaced the coil with a store-bought coil | 42.4 (37.2-47.7) | 23.8 (12.4-40.8) | 37.8 (18.7-61.5) | 37.0 (27.8-47.2) | 34.0 (20.0-51.5) | .7716 | |
| Replaced the coil with a coil someone else made | 36.4 (31.5-41.7) | 17.5 (8.1-33.6) | 26.0 (10.9-50.4) | 14.7 (9.2-22.7) | 2.0 (0.3-12.8) | <.0001 | |
| Changed the number of coils | 33.9 (29.1-39.1) | 7.8 (2.0-26.1) | 30.3 (13.8-54.2) | 20.7 (13.9-29.6) | 6.6 (2.1-19.0) | .0039 | |
| Changed the number of wraps to the coil | 33.3 (28.5-38.5) | 20.4 (10.0-37.2) | 16.7 (5.2-42.4) | 18.3 (12.0-27.0) | 4.6 (1.1-17.1) | .0060 | |
| Cleaned the coil | 49.0 (43.6-54.4) | 46.1 (30.4-62.5) | 53.8 (31.7-74.5) | 39.7 (30.3-50.0) | 48.2 (31.5-65.3) | .8408 | |
| Altered the coil in another way | 6.6 (4.4-10.1) | 6.0 (1.4-22.0) | 3.6 (0.5-22.2) | 7.8 (3.9-15.2) | 0 | .3441 | |
| **Young adults (18-29 years), n=634** |  |  |  |  |  |  | |
| Any coil modifications | 61.4 (54.9-67.6) | 72.0 (53.2-85.3) | 78.9 (58.2-91.0) | 54.7 (41.1-67.6) | 52.2 (27.0-76.3) | .2928 | |
| Built a coil | 22.5 (17.6-28.0) | 35.5 (17.8-58.5) | 21.3 (7.9-46.0) | 13.8 (7.5-24.0) | 3.4 (0.9-11.7) | .2917 | |
| Replaced the coil with a store-bought coil | 44.0 (37.8-50.3) | 43.7 (25.1-64.3) | 51.4 (28.0-74.2) | 39.3 (26.9-53.2) | 38.8 (17.9-64.7) | .9610 | |
| Replaced the coil with a coil someone else made | 18.3 (14.0-23.7) | 33.0 (15.9-56.1) | 23.3 (9.8-45.7) | 16.4 (8.6-29.2) | 25.4 (10.1-50.7) | .8156 | |
| Changed the number of coils | 18.9 (14.6-24.0) | 40.9 (22.9-61.7) | 31.8 (14.3-56.6) | 26.5 (16.5-39.7) | 11.8 (3.5-32.8) | .2850 | |
| Changed the number of wraps to the coil | 16.2 (12.3-21.0) | 22.6 (11.8-38.9) | 29.6 (12.7-54.8) | 25.2 (15.3-38.6) | 10.0 (2.5-32.3) | .6365 | |
| Cleaned the coil | 42.3 (36.2-48.6) | 42.0 (23.8-62.7) | 66.1 (43.0-83.4) | 33.2 (21.9-47.0) | 42.1 (20.6-67.0) | .1905 | |
| Altered the coil in another way | 4.5 (2.7-7.4) | 8.1 (2.7-22.0) | 3.7 (0.8-15.6) | 10.1 (4.3-22.0) | 2.7 (0.8-9.0) | .3608 | |
| **Older adults (30 years +), n=760** |  |  |  |  |  |  | |
| Any coil modifications | 56.8 (51.9-61.6) | 67.7 (49.8-81.6) | 65.4 (39.3-84.6) | 54.2 (39.4-68.3) | 44.2 (21.170.1) | .2253 | |
| Built a coil | 13.6 (10.1-18.1) | 30.2 (14.8-51.8) | 25.0 (8.5-54.6) | 21.9 (11.5-37.7) | 0 | .3125 | |
| Replaced the coil with a store-bought coil | 50.0 (44.9-55.0) | 52.5 (34.1-70.2) | 48.0 (24.6-72.3) | 37.3 (24.1-52.6) | 44.2 (21.1-70.1) | .9414 | |
| Replaced the coil with a coil someone else made | 8.1 (5.3-12.1) | 12.1 (4.8-31.0) | 16.8 (4.1-49.0) | 20.3 (10.3-36.0) | 4.5 (0.6-26.6) | .0748 | |
| Changed the number of coils | 9.5 (6.7-13.3) | 8.0 (2.4-23.6) | 11.9 (1.7-50.9) | 21.6 (11.8-36.2) | 1.9 (0.2-13.0) | .6063 | |
| Changed the number of wraps to the coil | 9.6 (6.7-13.5) | 15.4 (6.3-33.1) | 21.8 (6.5-52.6) | 18.2 (9.3-32.5) | 0 | .6067 | |
| Cleaned the coil | 27.1 (22.6-32.0) | 42.0 (24.8-61.4) | 55.0 (30.4-77.3) | 28.8 (17.6-43.5) | 15.2 (4.2-42.2) | .3799 | |
| Altered the coil in another way | 1.4 (0.6-2.9) | 4.8 (0.7-27.1) | 0 | 8.4 (2.6-24.5) | 0 | .0018 | |

**Supplementary Table 3. Weighted prevalence of battery modifications by race/ethnicity**

| **Battery modifications** | **Weighted % (95%CI)** | | | | | ***P* value** |
| --- | --- | --- | --- | --- | --- | --- |
|  | **White, Non-Hispanic** | **Black, Non-Hispanic** | **Other, Non-Hispanic** | **Hispanic** | **≥ 2 Races, Non-Hispanic** |  |
| **Youths (13-17 years), n=553** |  |  |  |  |  |  |
| Any battery modifications | 67.3 (61.9-72.3) | 44.9 (29.4-61.4) | 39.6 (20.5-62.6) | 55.0 (44.8-64.9) | 35.6 (21.3-53.0) | .0005 |
| Wired a new battery into the device | 53.8 (48.4-59.2) | 32.1 (18.9-48.9) | 12.9 (4.1-34.1) | 31.7 (23.2-41.6) | 7.3 (2.3-20.7) | <.0001 |
| Replaced the battery with an authorized replacement | 49.2 (43.8-54.6) | 29.2 (16.5-46.3) | 35.3 (17.3-58.8) | 36.7 (27.6-46.9) | 30.3 (17.2-47.6) | .0410 |
| Replaced the battery with a non-authorized replacement | 28.1 (23.6-33.0) | 21.0 (10.7-37.0) | 7.9 (1.9-27.4) | 17.9 (11.5-26.8) | 7.3 (2.3-20.7) | .0642 |
| Rewrapped a battery | 32.9 (28.1-38.0) | 14.0 (5.7-30.5) | 16.0 (4.9-41.4) | 24.8 (17.2-34.3) | 4.6 (1.1-17.1) | .0122 |
| Altered the battery in another way | 8.7 (6.1-12.3) | 9.8 (3.1-27.1) | 3.6 (0.5-22.2) | 9.4 (4.8-17.5) | 0 | .1686 |
| **Young adults (18-29 years), n=634** |  |  |  |  |  |  |
| Any battery modifications | 42.9 (36.7-49.3) | 65.1 (45.2-80.3) | 63.5 (40.9-81.4) | 43.1 (30.7-56.4) | 40.2 (19.5-65.1) | .1496 |
| Wired a new battery into the device | 16.7 (12.8-21.7) | 37.7 (20.2-59.2) | 30.6 (11.3-60.4) | 12.5 (7.1-20.9) | 20.4 (7.6-43.8) | .2814 |
| Replaced the battery with an authorized replacement | 30.2 (24.6-36.5) | 40.4 (22.1-61.8) | 48.4 (25.8-71.8) | 32.5 (21.7-45.6) | 34.5 (15.7-59.7) | .5476 |
| Replaced the battery with a non-authorized replacement | 9.4 (7.0-12.6) | 40.4 (22.4-61.4) | 3.9 (0.9-15.9) | 15.6 (8.4-27.0) | 17.8 (5.9-42.8) | .0030 |
| Rewrapped a battery | 11.8 (8.5-16.1) | 22.7 (11.7-39.4) | 7.5 (2.5-20.3) | 17.0 (9.3-29.1) | 9.2 (2.2-30.8) | .1185 |
| Altered the battery in another way | 6.9 (4.5-10.5) | 8.5 (3.0-21.8) | 3.9 (0.9-15.9) | 4.3 (1.6-10.9) | 3.0 (0.9-10.1) | .6727 |
| **Older adults (30 years +), n=760** |  |  |  |  |  |  |
| Any battery modifications | 33.5 (28.8-38.5) | 66.4 (49.2-80.1) | 37.9 (16.9-64.7) | 38.4 (25.1-53.8) | 44.1 (20.9-70.2) | .0130 |
| Wired a new battery into the device | 2.9 (1.7-4.9) | 20.6 (8.1-43.2) | 23.4 (7.6-53.2) | 15.2 (7.0-29.7) | 0 | <.0001 |
| Replaced the battery with an authorized replacement | 30.1 (25.6-35.0) | 61.6 (43.6-77.0) | 36.2 (15.6-63.6) | 37.0 (23.8-52.5) | 44.1 (20.9-70.2) | .0972 |
| Replaced the battery with a non-authorized replacement | 3.7 (2.2-6.1) | 18.4 (6.6-41.9) | 15.2 (3.2-49.2) | 20.1 (9.8-36.6) | 0 | .0003 |
| Rewrapped a battery | 4.3 (2.7-7.0) | 16.4 (5.7-38.8) | 20.2 (5.6-51.8) | 18.0 (8.8-33.3) | 9.4 (1.3-44.7) | .0203 |
| Altered the battery in another way | 0.8 (0.2-2.4) | 4.8 (0.7-27.1) | 6.6 (0.9-35.1) | 10.6 (3.8-26.1) | 0 | .0001 |

**Supplementary Table 4. Weighted prevalence of wattage/voltage adjustment by race/ethnicity**

| **Wattage/voltage adjustment** | **Weighted % (95%CI)** | | | | | ***P* value** |
| --- | --- | --- | --- | --- | --- | --- |
|  | **White, Non-Hispanic** | **Black, Non-Hispanic** | **Other, Non-Hispanic** | **Hispanic** | **≥ 2 Races, Non-Hispanic** |  |
| **Youths (13-17 years), n=553** |  |  |  |  |  |  |
| Any wattage/voltage adjustment | 51.5 (46.1-56.9) | 26.8 (14.6-43.9) | 27.6 (12.2-51.2) | 29.4 (21.0-39.4) | 47.5 (30.8-64.7) | .0006 |
| Used controls in the device to change wattage/voltage | 47.3 (42.0-52.7) | 22.9 (12.0-39.5) | 27.6 (12.2-51.2) | 24.3 (16.6-33.9) | 47.5 (30.8-64.7) | .0003 |
| Replaced the coil to have a different wattage/voltage | 32.6 (27.8-37.7) | 14 (5.7-30.5) | 16 (4.9-41.4) | 14.9 (9.0-23.6) | 3.9 (1.0-14.6) | <.0001 |
| Altered the device to change wattage/voltage | 30.5 (25.9-35.6) | 15.7 (6.9-32.0) | 11.7 (2.7-38.9) | 16.5 (10.3-25.3) | 4.6 (1.1-17.1) | .0098 |
| Altered the wattage/voltage in another way | 12.4 (9.3-16.3) | 13.7 (5.2-31.7) | 0 | 3.5 (1.3-9.2) | 0 | .0108 |
| **Young adults (18-29 years), n=634** |  |  |  |  |  |  |
| Any wattage/voltage adjustment | 50.7 (44.3-57.0) | 51.6 (32.4-70.4) | 57 (32.3-78.7) | 51.4 (38.0-64.5) | 40.2 (19.4-65.2) | .9180 |
| Used controls in the device to change wattage/voltage | 47.2 (40.9-53.7) | 33.7 (18.8-52.7) | 53.8 (29.9-76.1) | 44.4 (31.6-57.9) | 40.2 (19.4-65.2) | .7051 |
| Replaced the coil to have a different wattage/voltage | 24.8 (19.6-30.9) | 18.3 (8.8-34.2) | 32.2 (14.5-57.2) | 9.7 (5.4-16.7) | 30.3 (12.8-56.2) | .1655 |
| Altered the device to change wattage/voltage | 12.5 (9.1-17.0) | 7 (2.7-16.9) | 10 (4.1-22.4) | 16.7 (8.9-29.2) | 14.7 (4.3-39.8) | .9525 |
| Altered the wattage/voltage in another way | 4.1 (2.5-6.7) | 16.5 (4.0-48.6) | 1.1 (0.1-7.8) | 5.3 (2.0-13.5) | 2.2 (0.5-9.3) | .2827 |
| **Older adults (30 years +), n=760** |  |  |  |  |  |  |
| Any wattage/voltage adjustment | 53.4 (48.4-58.3) | 57.8 (39.7-74.1) | 48.2 (24.8-72.4) | 45.6 (31.4-60.4) | 44.6 (21.4-70.4) | .8042 |
| Used controls in the device to change wattage/voltage | 52.3 (47.3-57.3) | 57.8 (39.7-74.1) | 40 (18.6-65.9) | 44.8 (30.7-59.7) | 44.6 (21.4-70.4) | .6743 |
| Replaced the coil to have a different wattage/voltage | 19.4 (15.2-24.4) | 29.3 (13.8-51.8) | 11.5 (3.1-34.6) | 26.9 (15.3-42.9) | 13.5 (3.3-41.8) | .4617 |
| Altered the device to change wattage/voltage | 3.7 (2.2-6.0) | 17.6 (6.0-41.4) | 23.4 (7.6-53.2) | 13 (5.4-27.9) | 0 | <.0001 |
| Altered the wattage/voltage in another way | 1 (0.5-2.0) | 0 | 1.6 (0.2-11.2) | 11.6 (4.6-26.6) | 0 | <.0001 |

**Supplementary Table 5. Weighted prevalence of pod modification by race/ethnicity**

| **Pod modification** | **Weighted % (95%CI)** | | | | | ***P* value** |
| --- | --- | --- | --- | --- | --- | --- |
|  | **White, Non-Hispanic** | **Black, Non-Hispanic** | **Other, Non-Hispanic** | **Hispanic** | **≥ 2 Races, Non-Hispanic** |  |
| **Youths (13-17 years), n=553** |  |  |  |  |  |  |
| Any pod modifications | 60.8 (55.3-66.1) | 43.9 (28.6-60.5) | 32.7 (15.0-57.2) | 42.6 (33.0-52.8) | 24.4 (12.6-41.9) | .0001 |
| Refilled a pod not designed to be refilled | 46.8 (41.5-52.2) | 31.1 (18.2-47.8) | 16 (4.9-41.4) | 29.5 (21.2-39.4) | 11.6 (4.2-28.1) | .0008 |
| Cleaned the coil in a pod | 46.9 (41.6-52.3) | 36 (21.9-53.0) | 28.4 (12.0-53.7) | 27.8 (19.8-37.5) | 14.8 (6.4-30.6) | .0003 |
| Altered the pod in another way | 8 (5.5-11.6) | 6 (1.4-22.0) | 3.6 (0.5-22.2) | 6.1 (2.7-13.3) | 0 | .1944 |
| **Young adults (18-29 years), n=634** |  |  |  |  |  |  |
| Any pod modifications | 40.4 (34.4-46.8) | 54.3 (34.8-72.6) | 54.3 (30.8-76.0) | 45.8 (32.9-59.3) | 27.7 (12.0-51.8) | .3554 |
| Refilled a pod not designed to be refilled | 28 (22.7-34.0) | 38.8 (21.1-60.2) | 20.1 (8.7-39.8) | 28.2 (18.3-40.9) | 13.8 (4.9-33.4) | .3987 |
| Cleaned the coil in a pod | 22.9 (18.1-28.5) | 32.3 (15.4-55.4) | 47.4 (24.7-71.3) | 34.6 (22.8-48.6) | 25.9 (10.8-50.2) | .1137 |
| Altered the pod in another way | 3.1 (1.8-5.3) | 18.2 (5.1-48.1) | 1.1 (0.1-7.8) | 8.2 (3.1-19.9) | 1.7 (0.4-7.1) | .0488 |
| **Older adults (30 years +), n=760** |  |  |  |  |  |  |
| Any pod modifications | 14.8 (11.8-18.3) | 30.5 (16.2-49.9) | 52.4 (28.2-75.5) | 26.5 (15.6-41.3) | 5.9 (1.4-22.1) | .0001 |
| Refilled a pod not designed to be refilled | 9.2 (6.8-12.3) | 15.5 (7.0-30.8) | 21.8 (6.5-52.6) | 18.4 (9.4-32.9) | 0 | .1049 |
| Cleaned the coil in a pod | 7.8 (5.9-10.2) | 25.1 (12.0-45.2) | 52.4 (28.2-75.5) | 23.1 (12.9-37.8) | 5.9 (1.4-22.1) | <.0001 |
| Altered the pod in another way | 0.1 (0.0-0.7) | 0.9 (0.1-6.6) | 0 | 8.4 (2.5-24.4) | 0 | <.0001 |

**Supplementary Table 6. Weighted prevalence of building from scratch by race/ethnicity**

| **Building from scratch** | **Weighted % (95%CI)** | | | | | ***P* value** |
| --- | --- | --- | --- | --- | --- | --- |
|  | **White, Non-Hispanic** | **Black, Non-Hispanic** | **Other, Non-Hispanic** | **Hispanic** | **≥ 2 Races, Non-Hispanic** |  |
| **Youths (13-17 years), n=553** |  |  |  |  |  |  |
| Built from scratch | 26.2 (21.8-31.1) | 10.1 (3.6-25.1) | 11.7 (2.7-38.9) | 16.4 (10.2-25.2) | 4.6 (1.1-17.1) | .0573 |
| Used only parts designed by a manufacturer to be used in electronic vapor products | 24.8 (20.4-29.6) | 8.3 (2.6-23.8) | 11.7 (2.7-38.9) | 14.4 (8.7-23.0) | 4.6 (1.1-17.1) | .0543 |
| Used some electronic parts NOT designed for electronic vapor products | 20.7 (16.7-25.4) | 6 (1.4-22.0) | 3.6 (0.5-22.2) | 11.1 (6.3-18.9) | 4.6 (1.1-17.1) | .0451 |
| Used some other non-electronic household items NOT designed for electronic vapor devices | 19.1 (15.3-23.6) | 7.7 (2.3-22.7) | 3.6 (0.5-22.2) | 9.4 (5.0-17.0) | 4.6 (1.1-17.1) | .0070 |
| **Young adults (18-29 years), n=634** |  |  |  |  |  |  |
| Built from scratch | 8.3 (5.8-11.6) | 31.7 (15.1-54.7) | 3.5 (0.8-14.3) | 9.2 (4.7-17.2) | 3.9 (1.3-11.3) | .0003 |
| Used only parts designed by a manufacturer to be used in electronic vapor products | 6.7 (4.5-9.9) | 26.6 (11.1-51.2) | 3.5 (0.8-14.3) | 5.3 (2.6-10.7) | 3.9 (1.3-11.3) | .0009 |
| Used some electronic parts NOT designed for electronic vapor products | 5.6 (3.6-8.5) | 27.5 (11.8-51.9) | 3.5 (0.8-14.3) | 4 (1.7-9.0) | 1.3 (0.2-9.4) | <.0001 |
| Used some other non-electronic household items NOT designed for electronic vapor devices | 4.6 (3.0-7.2) | 25 (9.8-50.5) | 3.5 (0.8-14.3) | 4.5 (2.0-9.7) | 2.2 (0.5-9.3) | .0001 |
| **Older adults (30 years +), n=760** |  |  |  |  |  |  |
| Built from scratch | 1.2 (0.6-2.6) | 6.6 (1.7-21.7) | 15.2 (3.2-49.2) | 13.6 (5.9-28.4) | 0 | <.0001 |
| Used only parts designed by a manufacturer to be used in electronic vapor products | 1.2 (0.6-2.6) | 4.9 (0.9-21.6) | 15.4 (3.2-49.8) | 13.6 (5.7-28.8) | 0 | <.0001 |
| Used some electronic parts NOT designed for electronic vapor products | 1.2 (0.5-2.6) | 4.9 (0.9-21.6) | 15.4 (3.2-49.8) | 12.7 (5.1-28.3) | 0 | <.0001 |
| Used some other non-electronic household items NOT designed for electronic vapor devices | 0.6 (0.2-1.4) | 4.9 (0.9-21.6) | 12.1 (1.8-51.4) | 12.7 (5.1-28.3) | 0 | <.0001 |

**Supplementary Table 7. Weighted prevalence of reasons for e-liquid modifications**

| **Reasons for e-liquid modifications** | n | Enhance flavor | Save money | Use the same device longer | Increase cloud size | Decrease cloud size | Increase wattage/voltage | Decrease wattage/voltage | Make the battery last longer | Extend the life of the coil | Increase the nicotine hit | Decrease the nicotine hit | Try new things | Others I know do it | Heard/read about it | Make it safer to vape | Other reasons not listed | **Average no of reasons endorsed*** |
| --- | --- | --- | --- | --- | --- | --- | --- | --- | --- | --- | --- | --- | --- | --- | --- | --- | --- | --- |
| **Youth (13-17 years old)** |  |  |  |  |  |  |  |  |  |  |  |  |  |  |  |  |  |  |
| Made my own e-liquid | 204 | 90.3 | 71.8 | 62.9 | 69.4 | 48.6 | 53.6 | 54.0 | 53.1 | 53.6 | 63.6 | 71.1 | 62.1 | 57.7 | 70.4 | 65.7 | 14.4 | 9.5 |
| Mixed two or more purchased liquids together | 243 | 88.6 | 57.1 | 57.5 | 50.4 | 32.6 | 47.2 | 39.6 | 46.7 | 41.4 | 50.1 | 79.7 | 54.8 | 64.6 | 63.8 | 49.6 | 11.5 | 8.3 |
| Added nicotine to purchased e-liquid | 172 | 83.0 | 60.0 | 64.5 | 60.2 | 46.6 | 63.9 | 43.2 | 63.2 | 47.5 | 61.0 | 71.9 | 61.2 | 66.4 | 68.4 | 66.1 | 12.6 | 9.5 |
| Added flavorings to a purchased e-liquid | 262 | 91.2 | 52.2 | 60.0 | 51.8 | 35.0 | 44.0 | 42.5 | 45.0 | 40.6 | 46.7 | 78.0 | 56.7 | 64.7 | 63.6 | 54.5 | 9.2 | 8.5 |
| Added cannabis to a purchased e-liquid | 160 | 76.0 | 50.7 | 47.2 | 47.3 | 37.2 | 42.7 | 38.3 | 50.6 | 36.0 | 77.9 | 72.2 | 47.1 | 66.8 | 66.9 | 47.7 | 16.8 | 9.2 |
| Added other substances to a purchased e-liquid | 132 | 87.3 | 64.2 | 63.9 | 61.3 | 43.8 | 52.0 | 48.1 | 49.0 | 43.1 | 60.6 | 74.0 | 55.5 | 60.4 | 55.9 | 67.5 | 20.2 | 9.3 |
| Modified purchased e-liquids in another way | 64 | 86.9 | 65.8 | 64.7 | 57.9 | 58.9 | 54.1 | 58.3 | 55.6 | 63.3 | 52.3 | 65.1 | 53.6 | 60.8 | 64.1 | 67.5 | 32.4 | 10.9 |
| **Young adults (18-29 years)** |  |  |  |  |  |  |  |  |  |  |  |  |  |  |  |  |  |  |
| Made my own e-liquid | 107 | 72.4 | 68.8 | 55.3 | 54.6 | 31.5 | 44.3 | 37.5 | 45.9 | 43.3 | 44.0 | 69.4 | 60.0 | 46.1 | 51.9 | 57.6 | 21.4 | 8.0 |
| Mixed two or more purchased liquids together | 258 | 79.1 | 43.3 | 46.6 | 26.1 | 12.8 | 38.6 | 20.8 | 30.9 | 16.3 | 23.0 | 64.7 | 24.1 | 31.1 | 27.2 | 20.2 | 12.4 | 5.4 |
| Added nicotine to purchased e-liquid | 141 | 61.3 | 49.5 | 43.4 | 42.6 | 29.5 | 68.7 | 33.2 | 64.7 | 33.3 | 30.9 | 51.7 | 37.5 | 38.6 | 40.7 | 40.6 | 17.1 | 6.9 |
| Added flavorings to a purchased e-liquid | 185 | 81.7 | 45.4 | 54.0 | 38.2 | 25.6 | 35.8 | 28.9 | 37.2 | 24.5 | 32.0 | 59.8 | 40.3 | 39.0 | 38.7 | 32.4 | 11.2 | 6.4 |
| Added cannabis to a purchased e-liquid | 168 | 60.4 | 36.7 | 46.9 | 29.4 | 27.9 | 24.0 | 21.2 | 31.8 | 26.9 | 67.5 | 67.2 | 34.6 | 46.8 | 43.8 | 38.8 | 16.5 | 8.0 |
| Added other substances to a purchased e-liquid | 80 | 64.2 | 44.5 | 64.0 | 46.6 | 39.6 | 43.3 | 48.3 | 40.6 | 34.1 | 68.5 | 59.4 | 39.4 | 48.8 | 44.1 | 44.5 | 25.5 | 7.5 |
| Modified purchased e-liquids in another way | 41 | 86.1 | 58.6 | 61.8 | 63.8 | 59.4 | 38.2 | 72.5 | 50.2 | 50.9 | 47.0 | 61.0 | 63.6 | 64.6 | 63.3 | 80.2 | 38.0 | 10.4 |
| **Older adults (30 years +)** |  |  |  |  |  |  |  |  |  |  |  |  |  |  |  |  |  |  |
| Made my own e-liquid | 63 | 60.9 | 81.7 | 62.7 | 33.1 | 29.2 | 38.2 | 46.2 | 30.1 | 35.7 | 31.8 | 48.0 | 61.0 | 39.4 | 44.4 | 52.2 | 13.3 | 7.3 |
| Mixed two or more purchased liquids together | 248 | 76.1 | 38.2 | 51.8 | 10.0 | 7.0 | 29.6 | 24.3 | 19.3 | 13.9 | 16.3 | 52.9 | 16.4 | 18.5 | 16.5 | 10.4 | 14.3 | 4.2 |
| Added nicotine to purchased e-liquid | 95 | 40.4 | 43.2 | 38.4 | 16.0 | 16.5 | 77.9 | 24.5 | 41.9 | 17.7 | 22.6 | 36.8 | 23.8 | 25.6 | 18.7 | 16.9 | 9.3 | 5.0 |
| Added flavorings to a purchased e-liquid | 98 | 88.1 | 45.5 | 59.1 | 16.3 | 15.1 | 27.5 | 23.7 | 27.2 | 16.0 | 23.4 | 49.2 | 26.7 | 28.0 | 27.5 | 24.5 | 11.8 | 5.2 |
| Added cannabis to a purchased e-liquid | 82 | 67.9 | 37.1 | 29.1 | 21.6 | 20.0 | 20.7 | 25.6 | 24.5 | 23.8 | 74.4 | 54.5 | 32.5 | 43.9 | 43.5 | 29.1 | 15.8 | 7.7 |
| Added other substances to a purchased e-liquid | 29 | 72.2 | 49.2 | 67.5 | 32.0 | 48.7 | 27.6 | 45.9 | 43.0 | 48.9 | 67.4 | 73.3 | 60.7 | 62.7 | 49.6 | 39.1 | 26.3 | 8.3 |
| Modified purchased e-liquids in another way | 33 | 48.9 | 49.7 | 55.0 | 25.5 | 27.3 | 18.2 | 27.4 | 22.4 | 32.5 | 48.7 | 55.6 | 35.4 | 32.5 | 30.4 | 32.2 | 21.0 | 6.9 |

Note: * - average number of reasons endorsed was calculated only among people who made the specific modification and endorsed at least one reason for it.

**Supplementary Table 8. Weighted prevalence of reasons for coil modifications**

| **Reasons for coil modifications** | n | Enhance flavor | Save money | Use device longer | Increase cloud size | Decrease cloud size | Increase wattage/ voltage | Decrease wattage/ voltage | Make battery last longer | Extend the life of the coil | Increase nicotine hit | Decrease nicotine hit | Try new things | Others do it | Heard/read about it | Make it safer to vape | Other reasons | **Average no of reasons endorsed** |
| --- | --- | --- | --- | --- | --- | --- | --- | --- | --- | --- | --- | --- | --- | --- | --- | --- | --- | --- |
| **Youth (13-17 years old)** |  |  |  |  |  |  |  |  |  |  |  |  |  |  |  |  |  |  |
| Built a coil | 184 | 92.7 | 71.2 | 69.7 | 57.5 | 54.1 | 55.2 | 56.3 | 58.6 | 73.3 | 54.6 | 50.0 | 66.9 | 68.1 | 63.9 | 65.7 | 14.8 | 10.4 |
| Replaced the coil with a store-bought coil | 232 | 81.1 | 63.2 | 72.9 | 50.8 | 35.5 | 45.8 | 39.7 | 58.5 | 57.9 | 42.5 | 39.9 | 60.4 | 60.0 | 59.2 | 59.7 | 12.9 | 8.9 |
| Replaced the coil with a coil someone else made | 170 | 82.5 | 64.3 | 77.2 | 56.8 | 52.7 | 62.2 | 51.5 | 63.1 | 65.6 | 55.4 | 49.9 | 65.3 | 62.9 | 64.8 | 60.5 | 22.5 | 10.3 |
| Changed the number of coils | 166 | 83.8 | 64.9 | 61.7 | 58.2 | 48.1 | 61.9 | 46.4 | 54.2 | 60.9 | 61.8 | 53.0 | 67.7 | 58.2 | 54.7 | 61.3 | 17.4 | 9.8 |
| Changed the number of wraps to the coil | 162 | 88.1 | 65.8 | 73.1 | 60.6 | 54.9 | 57.5 | 58.8 | 61.1 | 65.9 | 56.7 | 58.9 | 66.4 | 63.9 | 68.2 | 63.3 | 20.9 | 10.4 |
| Cleaned the coil | 269 | 78.2 | 65.3 | 72.5 | 47.3 | 35.0 | 46.4 | 36.0 | 59.6 | 63.4 | 43.4 | 36.2 | 55.8 | 56.3 | 58.1 | 52.2 | 12.2 | 8.7 |
| Altered the coil in another way | 34 | 83.0 | 71.7 | 66.0 | 63.9 | 72.2 | 68.1 | 69.9 | 69.1 | 76.9 | 62.1 | 57.1 | 79.6 | 62.3 | 62.1 | 58.2 | 59.7 | 11.6 |
| **Young adults (18-29 years)** |  |  |  |  |  |  |  |  |  |  |  |  |  |  |  |  |  |  |
| Built a coil | 150 | 68.8 | 60.6 | 64.6 | 53.6 | 38.5 | 44.9 | 38.5 | 33.3 | 55.0 | 31.9 | 31.6 | 46.9 | 49.7 | 46.1 | 32.3 | 21.5 | 7.5 |
| Replaced the coil with a store-bought coil | 287 | 65.9 | 41.2 | 71.7 | 34.2 | 13.4 | 39.7 | 16.1 | 35.9 | 47.7 | 24.9 | 13.9 | 27.9 | 35.2 | 34.2 | 40.2 | 12.3 | 5.9 |
| Replaced the coil with a coil someone else made | 131 | 70.8 | 64.7 | 65.0 | 49.1 | 42.1 | 56.5 | 38.2 | 44.1 | 51.0 | 37.9 | 27.5 | 54.4 | 46.8 | 40.3 | 33.2 | 16.6 | 7.9 |
| Changed the number of coils | 163 | 69.4 | 53.5 | 51.9 | 57.7 | 39.2 | 56.0 | 37.5 | 45.7 | 47.2 | 47.0 | 28.7 | 55.3 | 52.4 | 55.6 | 42.8 | 13.8 | 8.4 |
| Changed the number of wraps to the coil | 138 | 65.7 | 44.9 | 50.3 | 46.7 | 37.2 | 49.3 | 35.6 | 31.5 | 47.9 | 34.6 | 31.8 | 50.6 | 42.1 | 50.3 | 35.7 | 11.9 | 7.5 |
| Cleaned the coil | 284 | 66.7 | 65.0 | 72.2 | 29.9 | 18.6 | 29.4 | 17.5 | 34.4 | 64.7 | 27.3 | 17.1 | 33.0 | 39.7 | 46.3 | 39.1 | 8.3 | 6.7 |
| Altered the coil in another way | 43 | 55.1 | 40.2 | 40.9 | 32.4 | 44.1 | 33.2 | 43.2 | 43.1 | 51.1 | 46.8 | 50.1 | 57.7 | 57.5 | 49.9 | 49.8 | 38.1 | 8.8 |
| **Older adults (30 years +)** |  |  |  |  |  |  |  |  |  |  |  |  |  |  |  |  |  |  |
| Built a coil | 82 | 56.1 | 73.9 | 65.7 | 41.1 | 19.1 | 32.6 | 25.6 | 29.4 | 58.9 | 22.4 | 25.4 | 59.0 | 41.5 | 48.4 | 26.5 | 11.5 | 6.8 |
| Replaced the coil with a store-bought coil | 337 | 57.1 | 21.7 | 66.9 | 12.9 | 5.6 | 13.7 | 7.9 | 14.5 | 33.9 | 13.5 | 5.9 | 16.0 | 13.5 | 15.7 | 19.3 | 12.8 | 3.5 |
| Replaced the coil with a coil someone else made | 71 | 73.7 | 69.4 | 62.7 | 35.3 | 30.6 | 30.3 | 23.9 | 25.9 | 44.8 | 39.4 | 26.4 | 56.5 | 52.6 | 40.5 | 36.3 | 18.8 | 7.1 |
| Changed the number of coils | 58 | 70.1 | 45.6 | 52.8 | 44.2 | 28.0 | 55.2 | 31.5 | 37.4 | 49.4 | 27.1 | 24.9 | 53.6 | 39.1 | 42.1 | 31.2 | 11.5 | 7.6 |
| Changed the number of wraps to the coil | 63 | 74.3 | 45.1 | 48.9 | 53.8 | 28.6 | 44.8 | 42.9 | 42.1 | 59.5 | 25.9 | 32.4 | 56.1 | 42.1 | 43.4 | 31.4 | 13.4 | 7.3 |
| Cleaned the coil | 186 | 82.3 | 69.4 | 75.2 | 19.2 | 8.5 | 17.3 | 8.5 | 20.3 | 68.6 | 19.8 | 8.4 | 18.3 | 24.1 | 29.8 | 24.0 | 6.6 | 5.3 |
| Altered the coil in another way | 12 | 90.6 | 57.3 | 81.0 | 58.4 | 77.4 | 52.6 | 66.6 | 52.8 | 81.0 | 63.7 | 67.6 | 58.4 | 79.9 | 58.4 | 58.5 | 59.7 | 11.7 |

**Supplementary Table 9. Weighted prevalence of reasons for battery modifications**

| **Reasons for battery modifications** | n | Enhance flavor | Save money | Increase cloud size | Decrease cloud size | Increase wattage/voltage | Decrease wattage/voltage | Make the battery last longer | Build electronic vapor products from scratch | Increase the nicotine hit | Decrease the nicotine hit | Try new things | Others I know do it | Heard/read about it | Other reasons not listed | **Average no of reasons endorsed** |
| --- | --- | --- | --- | --- | --- | --- | --- | --- | --- | --- | --- | --- | --- | --- | --- | --- |
| **Youth (13-17 years old)** |  |  |  |  |  |  |  |  |  |  |  |  |  |  |  |  |
| Wired a new battery into the device | 258 | 81.3 | 66.9 | 60.6 | 36.3 | 62.6 | 44.9 | 65.3 | 55.3 | 56.3 | 45.4 | 63.2 | 52.7 | 58.1 | 14.2 | 7.9 |
| Replaced the battery with an authorized replacement | 253 | 66.7 | 64.7 | 53.3 | 34.7 | 52.7 | 36.2 | 59.4 | 43.6 | 40.8 | 36.6 | 52.1 | 51.9 | 53.0 | 13.2 | 6.9 |
| Replaced the battery with a non-authorized replacement | 142 | 80.4 | 69.6 | 67.4 | 49.6 | 65.5 | 48.5 | 65.8 | 65.0 | 60.5 | 52.3 | 70.7 | 60.3 | 66.5 | 18.6 | 8.4 |
| Rewrapped a battery | 163 | 79.5 | 70.4 | 64.7 | 55.2 | 64.8 | 51.2 | 68.6 | 67.5 | 60.4 | 52.9 | 68.9 | 63.1 | 75.8 | 18.6 | 8.7 |
| Altered the battery in another way | 44 | 85.5 | 67.5 | 69.2 | 52.8 | 73.7 | 56.3 | 63.0 | 55.8 | 70.2 | 37.6 | 61.2 | 47.5 | 81.9 | 52.2 | 9.1 |
| **Young adults (18-29 years)** |  |  |  |  |  |  |  |  |  |  |  |  |  |  |  |  |
| Wired a new battery into the device | 142 | 65.1 | 65.4 | 53.2 | 36.8 | 50.6 | 36.3 | 61.3 | 46.6 | 47.4 | 37.7 | 55.8 | 47.9 | 54.3 | 22.7 | 6.9 |
| Replaced the battery with an authorized replacement | 218 | 35.9 | 50.0 | 32.5 | 17.1 | 40.4 | 15.6 | 63.2 | 23.0 | 29.6 | 13.9 | 29.3 | 32.2 | 32.5 | 11.7 | 4.6 |
| Replaced the battery with a non-authorized replacement | 107 | 73.0 | 57.2 | 62.4 | 42.6 | 58.2 | 47.6 | 58.9 | 54.3 | 50.8 | 45.0 | 59.2 | 49.3 | 56.1 | 31.3 | 7.8 |
| Rewrapped a battery | 102 | 49.8 | 60.9 | 51.1 | 39.2 | 51.8 | 32.4 | 64.6 | 43.4 | 47.1 | 33.9 | 46.6 | 51.2 | 65.2 | 31.3 | 6.7 |
| Altered the battery in another way | 48 | 72.5 | 39.5 | 65.2 | 32.4 | 56.9 | 36.1 | 49.9 | 39.9 | 48.4 | 40.9 | 65.9 | 57.7 | 51.2 | 48.9 | 7.6 |
| **Older adults (30 years +)** |  |  |  |  |  |  |  |  |  |  |  |  |  |  |  |  |
| Wired a new battery into the device | 55 | 69.0 | 60.2 | 58.3 | 44.2 | 70.7 | 60.9 | 70.8 | 48.1 | 58.6 | 49.4 | 72.9 | 55.3 | 65.2 | 21.1 | 8.0 |
| Replaced the battery with an authorized replacement | 220 | 19.9 | 34.1 | 11.6 | 7.9 | 21.2 | 10.7 | 49.1 | 7.7 | 13.6 | 6.9 | 12.9 | 12.7 | 14.7 | 22.9 | 2.8 |
| Replaced the battery with a non-authorized replacement | 35 | 54.9 | 82.2 | 51.6 | 27.8 | 66.3 | 37.8 | 62.2 | 43.6 | 47.9 | 34.8 | 44.1 | 44.0 | 47.8 | 17.7 | 6.6 |
| Rewrapped a battery | 41 | 44.7 | 59.1 | 40.3 | 29.9 | 45.2 | 33.1 | 80.9 | 27.1 | 39.1 | 31.8 | 40.2 | 41.6 | 43.3 | 26.0 | 6.0 |
| Altered the battery in another way | 10 | 75.6 | 81.1 | 64.0 | 81.1 | 75.6 | 69.5 | 78.6 | 66.5 | 90.2 | 55.2 | 78.6 | 43.7 | 90.2 | 61.8 | 11.2 |

**Supplementary Table 10. Weighted prevalence of reasons for wattage/voltage modifications**

| **Reasons for wattage/voltage modifications** | n | Enhance flavor | Save money | Increase cloud size | Decrease cloud size | Make the battery last longer | Increase power going to the coil | Decrease power going to the coil | Build electronic vapor products from scratch | Increase the nicotine hit | Decrease the nicotine hit | Try new things | Because others I know do it | Heard/read about it | Make vaping safer | Other reasons not listed | **Average no of reasons endorsed** |
| --- | --- | --- | --- | --- | --- | --- | --- | --- | --- | --- | --- | --- | --- | --- | --- | --- | --- |
| **Youth (13-17 years old)** |  |  |  |  |  |  |  |  |  |  |  |  |  |  |  |  |  |
| Used controls in the device to change wattage/voltage | 234 | 71.8 | 54.9 | 61.6 | 40.1 | 60.2 | 59.6 | 45.4 | 45.5 | 53.5 | 44.4 | 57.6 | 53.3 | 52.8 | 52.3 | 10.2 | 8.0 |
| Replaced the coil to have a different wattage/voltage | 149 | 80.2 | 70.9 | 66.2 | 49.4 | 65.1 | 64.5 | 50.1 | 60.9 | 56.0 | 47.5 | 63.9 | 57.2 | 61.2 | 64.6 | 19.3 | 9.1 |
| Altered the device to change wattage/voltage | 143 | 82.3 | 66.9 | 65.3 | 46.2 | 67.4 | 57.4 | 51.8 | 54.8 | 61.4 | 50.3 | 60.6 | 59.4 | 69.4 | 61.3 | 16.2 | 9.2 |
| Altered the wattage/voltage in another way | 54 | 91.0 | 71.4 | 73.5 | 58.2 | 59.5 | 59.3 | 67.3 | 72.2 | 67.7 | 67.3 | 65.7 | 70.8 | 53.9 | 63.5 | 36.3 | 10.4 |
| **Young adults (18-29 years)** |  |  |  |  |  |  |  |  |  |  |  |  |  |  |  |  |  |
| Used controls in the device to change wattage/voltage | 299 | 56.9 | 27.7 | 66.7 | 31.6 | 49.8 | 60.3 | 49.1 | 12.4 | 48.4 | 29.2 | 38.8 | 34.3 | 34.7 | 23.9 | 6.8 | 6.0 |
| Replaced the coil to have a different wattage/voltage | 148 | 71.3 | 37.1 | 68.1 | 34.8 | 38.5 | 58.8 | 39.8 | 24.1 | 42.8 | 31.7 | 44.5 | 35.9 | 40.8 | 27.8 | 7.8 | 6.5 |
| Altered the device to change wattage/voltage | 100 | 73.8 | 41.9 | 53.3 | 39.9 | 43.2 | 43.9 | 43.7 | 30.5 | 46.1 | 27.8 | 45.1 | 31.1 | 34.5 | 28.3 | 19.2 | 6.5 |
| Altered the wattage/voltage in another way | 38 | 86.0 | 60.4 | 66.9 | 70.9 | 42.0 | 45.7 | 51.6 | 41.9 | 31.0 | 43.8 | 61.1 | 65.6 | 63.7 | 52.3 | 46.3 | 9.1 |
| **Older adults (30 years +)** |  |  |  |  |  |  |  |  |  |  |  |  |  |  |  |  |  |
| Used controls in the device to change wattage/voltage | 339 | 61.1 | 13.4 | 45.1 | 24.6 | 36.8 | 60.8 | 49.8 | 5.3 | 24.5 | 14.4 | 21.9 | 14.3 | 14.7 | 13.6 | 5.9 | 4.2 |
| Replaced the coil to have a different wattage/voltage | 104 | 73.7 | 37.7 | 40.3 | 26.0 | 53.5 | 51.4 | 39.9 | 10.6 | 26.2 | 17.5 | 30.3 | 21.7 | 21.4 | 18.4 | 5.6 | 5.1 |
| Altered the device to change wattage/voltage | 37 | 68.5 | 38.5 | 50.1 | 35.7 | 57.6 | 66.8 | 61.0 | 45.3 | 60.2 | 48.5 | 47.9 | 47.5 | 47.9 | 55.7 | 18.3 | 7.9 |
| Altered the wattage/voltage in another way | 15 | 86.7 | 68.5 | 68.1 | 42.7 | 68.9 | 64.8 | 56.0 | 51.6 | 66.6 | 54.4 | 71.9 | 70.1 | 76.3 | 74.9 | 41.8 | 9.6 |

**Supplementary Table 11. Weighted prevalence of reasons for pod modifications**

| **Reasons for pod modifications** | n | Enhance flavor | Try other flavors | Save money | Increase cloud size | Decrease cloud size | Increase nicotine level | Decrease nicotine level | Add marijuana or cannabis | Extend the life of the pod | Customize the look of my device | Increase the nicotine hit | Decrease the nicotine hit | Try new things | Others I know do it | Heard/read about it | Make it safer to vape | Other reasons not listed | **Average no of reasons endorsed** |
| --- | --- | --- | --- | --- | --- | --- | --- | --- | --- | --- | --- | --- | --- | --- | --- | --- | --- | --- | --- |
| **Youth (13-17 years old)** |  |  |  |  |  |  |  |  |  |  |  |  |  |  |  |  |  |  |  |
| Refilled a pod not designed to be refilled | 229 | 80.4 | 62.8 | 69.8 | 53.7 | 50.3 | 45.3 | 45.1 | 53.9 | 61.6 | 55.9 | 53.5 | 48.3 | 66.6 | 57.1 | 61.6 | 76.9 | 50.4 | 9.6 |
| Cleaned the coil in a pod | 233 | 79.2 | 70.7 | 68.6 | 52.7 | 39.9 | 51.5 | 44.8 | 43.1 | 66.2 | 57.1 | 49.0 | 45.9 | 64.6 | 58.5 | 65.2 | 76.9 | 50.4 | 9.6 |
| Altered the pod in another way | 37 | 85.6 | 69.7 | 71.2 | 68.5 | 62.4 | 66.7 | 57.7 | 65.9 | 62.6 | 76.9 | 76.2 | 56.4 | 82.9 | 72.8 | 80.5 | 76.9 | 50.4 | 11.8 |
| **Young adults (18-29 years)** |  |  |  |  |  |  |  |  |  |  |  |  |  |  |  |  |  |  |  |
| Refilled a pod not designed to be refilled | 199 | 48.4 | 47.8 | 74.0 | 33.8 | 27.3 | 35.1 | 34.6 | 34.5 | 61.1 | 28.9 | 38.3 | 22.6 | 48.5 | 40.9 | 36.4 | 27.2 | 16.6 | 7.0 |
| Cleaned the coil in a pod | 176 | 54.9 | 48.0 | 57.3 | 30.5 | 21.2 | 26.9 | 24.7 | 31.0 | 54.8 | 22.8 | 31.5 | 21.2 | 44.9 | 38.4 | 42.5 | 34.5 | 8.5 | 6.5 |
| Altered the pod in another way | 35 | 76.2 | 43.9 | 85.7 | 50.5 | 64.9 | 44.1 | 63.0 | 48.7 | 59.4 | 44.1 | 72.6 | 48.5 | 60.3 | 50.1 | 69.3 | 50.8 | 56.0 | 9.9 |
| **Older adults (30 years +)** |  |  |  |  |  |  |  |  |  |  |  |  |  |  |  |  |  |  |  |
| Refilled a pod not designed to be refilled | 84 | 40.2 | 46.9 | 83.8 | 26.8 | 20.6 | 24.2 | 27.6 | 31.8 | 63.7 | 28.3 | 29.6 | 27.5 | 36.2 | 36.7 | 49.9 | 30.6 | 13.0 | 6.7 |
| Cleaned the coil in a pod | 95 | 69.2 | 44.9 | 52.3 | 23.9 | 18.6 | 28.2 | 22.6 | 27.5 | 45.9 | 25.1 | 29.0 | 16.6 | 35.6 | 37.3 | 38.4 | 37.1 | 14.3 | 5.8 |
| Altered the pod in another way | 5 | 100.0 | 86.2 | 100.0 | 78.9 | 100.0 | 78.9 | 64.5 | 50.7 | 71.7 | 86.2 | 92.8 | 50.7 | 100.0 | 57.9 | 92.8 | 78.9 | 92.8 | 13.8 |

**Supplementary Table 12. Weighted prevalence of reasons for building from scratch**

| **Reasons for building from scratch** | n | Enhance flavor | Save money | Increase cloud size | Decrease cloud size | Increase wattage/voltage | Decrease wattage/voltage | Make the battery last longer | Increase the nicotine hit | Decrease the nicotine hit | Try new things | Because Others I know do it. | Heard/read about it | Make a product that is more durable | Make a product that is safer | Other reasons not listed | **Average no of reasons endorsed** |
| --- | --- | --- | --- | --- | --- | --- | --- | --- | --- | --- | --- | --- | --- | --- | --- | --- | --- |
| **Youth (13-17 years old)** | 119 | 81.5 | 73.8 | 64.3 | 50.3 | 61.2 | 47.9 | 69.1 | 50.8 | 63.4 | 66.7 | 59.5 | 63.9 | 72.8 | 61.9 | 15.1 | 9.2 |
| **Young adults (18-29 years)** | 77 | 80.4 | 61.3 | 52.3 | 45.8 | 63.6 | 51.9 | 57.5 | 46.4 | 48.1 | 59.9 | 64.8 | 59.8 | 67.4 | 48.6 | 33.7 | 8.5 |
| **Older adults (30 years +)** | 19 | 90.5 | 83.8 | 87.5 | 48.8 | 82.6 | 60.7 | 81.2 | 65.5 | 87.5 | 68.3 | 84.6 | 74.9 | 86.3 | 71.0 | 48.9 | 11.7 |
